# Effects of LED photoperiods and light qualities on in vitro growth and chlorophyll fluorescence of *Cunninghamia lanceolata*

**DOI:** 10.1186/s12870-020-02480-7

**Published:** 2020-06-09

**Authors:** Yuanyuan Xu, Mei Yang, Fei Cheng, Shinan Liu, Yuyao Liang

**Affiliations:** 1 Guangxi Key Laboratory of Forest Ecology and Conservation, College of Forestry, Guangxi University, Nanning, 530004 Guangxi PR China; 2 College of Forestry, Beijing Forestry University, Beijing, 100083 PR China; 3 College of Resources and Environment, Fujian Agriculture and Forestry University, Fuzhou, 350002 Fujian PR China

**Keywords:** Light emitting diodes (LEDs), Photoperiod, Light quality, *Cunninghamia lanceolata*, Growth, Chlorophyll fluorescence

## Abstract

**Background:**

*Cunninghamia lanceolata (C. lanceolata)* is the main fast-growing timber species in southern China. As an alternative to conventional lighting systems, LED has been demonstrated to be an artificial flexible lighting source for commercial micropropagation. The application of LED can provide rapid propagation of *C. lanceolata* in vitro culture.

**Results:**

We applied two-factor randomized block design to study the effects of LED photoperiods and light qualities on the growth and chlorophyll fluorescence of *C. lanceolata* in vitro culture plantlets. In this study, plantlets were exposed to 20 μmol·m^− 2^·s^− 1^ irradiance for three photoperiods, 8, 16, and 24 h under the three composite lights, 88.9% red+ 11.1% blue (R/B), 80.0% red+ 10.0% blue+ 10.0% purple (R/B/P), 72.7% red+ 9.1% blue+ 9.1% purple+ 9.1% green (R/B/P/G), as well as white light (12.7% red+ 3.9% blue+ 83.4% green, W) as control. The results showed that: (1) Plant height, dry weight, rooting rate, average root number, length, surface area and volume, chlorophyll, and chlorophyll fluorescence parameters were significantly affected by photoperiods, light qualities and their interactions. (2) Plantlets subjected to photoperiod 16 h had longer root, higher height, rooting rate, root number, and the higher levels of chlorophyll, chlorophyll a/b, Y (II), qP, NPQ/4 and ETR_II_ compared to photoperiods 8 h and 24 h, while Fv/Fm during photoperiod 16 h was lower than 8 h and 24 h. Plantlets exposed to R/B/P/G generated more root and presented higher chlorophyll, Fv/Fo, Y (II), qP, and ETR_II_ than W during photoperiods 8 and 16 h. (3) Total chlorophyll content and ETR_II_ were significant correlated with rooting rate, root length and root volume, while Fv/Fm and ETR_II_ were significant correlated with plant height, average root number and root surface area. (4) 16-R/B/P/G is best for growing *C. lanceolata* plantlets in vitro.

**Conclusions:**

This study demonstrated the effectiveness of photoperiods and light qualities using LEDs for micropropagation of *C. lanceolata*. The best plantlets were harvested under 16-R/B/P/G treatment. And there was a correlation between the growth and the chlorophyll and chlorophyll fluorescence of their leaves under different photoperiod and light quality. These results can contribute to improve the micropropagation process of this species.

## Background

As a signal and energy source, light is one the most important environment factors for plant growth and development in commercial micropropagation laboratories. Light quality (spectral quality), photoperiod and quantity (photon flux) have a profound influence on the morphogenesis and growth of plant cell, tissue and organ cultures [1]. As an alternative to conventional light sources for in vitro plant growth, light-emitting diodes (LEDs) have emerged with several unique advantages, including customizable spectra, small mass and volume, energy consumption, lower radiative heat emission, adjustable light intensity, longer lifetime, and high energy-conversion efficiency [2, 3]. Therefore, LED is applied to plant facility cultivation to solve the problems of luminous efficiency, high heat dissipation, high energy consumption, and mixed spectrum if using traditional light sources such as high-pressure sodium lamps, fluorescent lamps, metal halide lamps, and incandescent lamps [3].

The flexibility of matching wavelengths of LEDs to plant photoreceptors may provide more optimal production, influencing plant morphology and physiology [2, 4]. The assessment of the effect of different LED wavelengths on the growth and development of in vitro plantlets of various plant species has drawn more attention [5]. However, the responses vary according to plant species and growth stages. Monochromatic red light can promote the germination of somatic embryos of *Pinus densiflora* [6]. Kwon et al. [7] pointed out that R/B (1:1) can promote cell division of *Populus euramericana* on in vitro culture than monochromatic light or fluorescence. Similarly, the biomass of stems and roots of *Vaccinium corymbosum* in vitro plantlets treated with R/B (1:1) were significantly higher than monochromatic red and blue light [8]. Compared to white light (W), *Eucalyptus*, *Musa*, and *Spathiphyllum* showed higher growth of plantlets grown in vitro under a combination of red and blue light (R/B, 4/1) [9]. Huang et al. [10] reported that R/B (5:5) could promote Apple Root-Stock JM7 germination in vitro, while R/B (6:4) was best to its proliferation and root growth. In the ‘Favorita’ potato (*Solanum tuberosum* L.) plantlets cultured in vitro, plant height was the greatest under monochromatic red light, whereas root length and fresh weight were the greatest under R/B (8/2), and chlorophyll content was the greatest under R/B (7/3) [11]. Cho et al. [12] pointed out that the combination of red and green light (R/G, 80/20) increased more than twice as much roots and dry mass compared with W in Coleus (*Plectranthus scutellarioides*) in vitro culture, and the exposure to the combination of red, green and blue light (R/G/B, 40/20/40) significantly increased the chlorophyll content. LED can influence the physiological process of plantlets cultured in vitro through the optimal combination of different light qualities, so as to promote the growth and improve the quality of plantlets.

*Cunninghamia lanceolata* (Lamb.) Hook. (Taxodiaceae, *Cunninghamia*) is one of the most popular timber tree species in architecture, furniture and other industries in China, because of its fast growth, soft material, straight texture and easy processing. It has been planted in China for more than 3000 years. At present, the planting area exceeds 11 million hectares, accounting for 20–30% of China’s total commercial timber output [13, 14]. Clonal forestry has been playing an increasingly important role in plantation cultivation of commercial wood in some region. Clonal afforestation can not only keep the parent’s excellent characteristics, but also establish a stable forest. In vitro regeneration plays a major role in the propagation of plant, producing a large number of plantlets with the identical characteristics as their parent plants. Recently, several in vitro regeneration protocols have been proposed for *C. lanceolata*, to improve biomass production of these rapidly growing, high quality trees, by selection of elite genotypes [15]. At present, the research on in vitro culture of *C. lanceolata* are mostly focused on the traditional methods, such as adjusting the basic medium and growth regulators, and its artificial light source is usually the traditional fluorescent lamp, which has the problem of slow growth of roots, resulting in low production efficiency and low economic benefit [15, 16]. The application of LED in this field is still rare, only in the following two studies. Ding et al. [17] showed that the growth rate of *C. lanceolata* in vitro plantlets could be increased by using monochromatic red light in subculture stage, and then using monochromatic blue light could promote root and plantlet growth in rooting stage. Zhou et al. [18] pointed out that compared with monochromatic blue light and green light, *C. lanceolata* in vitro plantlets under monochromatic red light had the highest chlorophyll a, chlorophyll b, total chlorophyll content, photochemical efficiency of photosystem II (PSII) (Fv/Fo) and maximum quantum yield of PSII (Fv/Fm). However, there is no report on the study considering both photoperiod and light quality at the same time in vitro culture of *C. lanceolata*. On the other hand, chlorophyll fluorescence is a sensitive probe for plant photosynthesis, which has been widely used in recent years. It can be used to measure and study the dynamic changes of photosynthesis and to detect the effects of various external factors on plant growth [19]. What we are interested in is how the response of growth and chlorophyll fluorescence of *C. lanceolata* in vitro plantlets to LED photoperiod and light quality. What is the best photoperiod and light quality for in vitro culture of *C. lanceolata*? Under the LED, is there a certain relationship between the growth and chlorophyll fluorescence? Therefore, the objectives of this study were: 1) the examination of the effects of LED compound light, photoperiod and their interaction on the growth and chlorophyll fluorescence of *C. lanceolata* in vitro tissue culture; 2) the determination of the best combination of photoperiod and light quality for in vitro culture of *C. lanceolata*; 3) to study the relationship between the growth and chlorophyll fluorescence. We look forward the results of this study might provide data reference for discussing the effect of LED on the micropropagation process of *C. lanceolata*, and help to optimize technical approach for large-scale production in vitro culture of this commercial tree species.

## Results

### Effects of photoperiods and light qualities on the growth

**Fig. 1:**
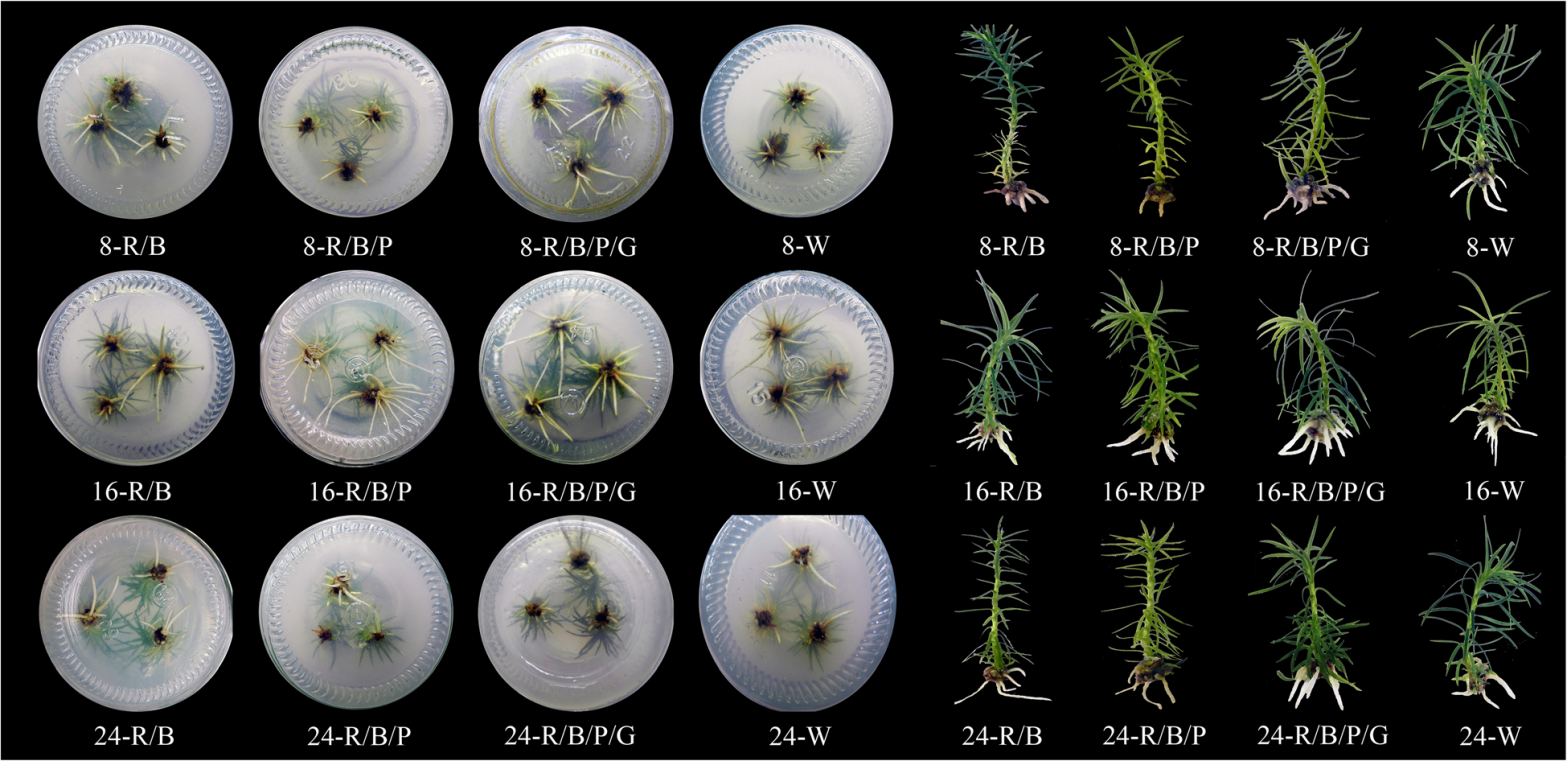
Growth of *C. lanceolata* in vitro culture plantlets under different photoperiods and light qualities. 8, 16 and 24 refer to photoperiod 8, 16, 24 h, respectively; R/B, R/B/P, R/B/P/G, and W refer to light quality R/B (88.9% red+ 11.1% blue), R/B/P (80.0% red+ 10.0% blue+ 10.0% purple), R/B/P/G (72.7% red+ 9.1% blue+ 9.1% purple+ 9.1% green), W (12.7% red+ 3.9% blue+ 83.4% green)

The growth of *C. lanceolata* in vitro plantlets was found to be significantly affected by the photoperiods, light qualities and their interactions. The height, dry weight, rooting rate, average root number, root length, root surface area, and volume were the highest at photoperiod 16 h at most light qualities (Table 1). And under this photoperiod, plantlets’ roots and leaves were numerous, long, and evenly distributed (Fig. 1). With the photoperiods of 8 and 16 h, height, rooting rate, root length, root surface area, and root volume were significantly higher in the R/B/P/G compared to those with R/B, R/B/P and W (*P* < 0.05). The dry weights were significantly enhanced in the R/B/P group for all three photoperiods compared to W treatment (*P *< 0.05). Rooting rate, root length, surface area, and volume reached the highest point with the 16-R/B/P/G treatment.

**Table 1:** Effects of photoperiods and light qualities on the growth of *C. lanceolata* in vitro culture plantlets

Photoperiod/ h·d^− 1^ (A)	Light quality^a^ (B)	Height/cm	Dry weight /mg	Rooting rate/%	Average root number	Root length/cm	Root surface area/cm^2^	Root volume/cm^3^
8	R/B	1.73 ± 0.05^b^ i^c^	23.07 ± 0.03 i	32.50 ± 1.50 h	0.51 ± 0.03 j	1.10 ± 0.02 k	0.33 ± 0.05 k	0.34 ± 0.02 i
	R/B/P	1.65 ± 0.03 j	34.61 ± 1.01 e	10.17 ± 0.93 i	0.20 ± 0.02 k	0.30 ± 0.01 l	0.08 ± 0.01 l	0.12 ± 0.01 j
	R/B/P/G	2.07 ± 0.06 g	29.21 ± 1.02 g	76.53 ± 2.83 c	2.06 ± 0.02 g	1.47 ± 0.01 i	0.47 ± 0.02 i	0.62 ± 0.02 g
	W	1.98 ± 0.03 h	23.15 ± 2.01 i	67.53 ± 1.23 e	2.47 ± 0.04 f	1.27 ± 0.22 j	0.46 ± 0.01 j	0.44 ± 0.05 h
16	R/B	2.40 ± 0.02 d	37.22 ± 3.01 c	72.70 ± 3.03 d	2.85 ± 0.03 e	2.52 ± 0.20 c	0.66 ± 0.01 e	0.78 ± 0.05 d
	R/B/P	2.44 ± 0.03 d	46.13 ± 3.42 a	65.83 ± 4.50 f	3.61 ± 0.03 d	3.85 ± 0.11 d	1.18 ± 0.05 c	0.80 ± 0.04 c
	R/B/P/G	2.63 ± 0.02 b	32.77 ± 2.12 f	95.50 ± 3.04 a	4.45 ± 0.04 b	5.95 ± 0.30 a	1.92 ± 0.07 a	1.02 ± 0.02 a
	W	2.41 ± 0.02 d	20.53 ± 0.93 j	86.60 ± 3.51 b	4.63 ± 0.05 a	4.71 ± 0.50 b	1.49 ± 0.03 b	1.02 ± 0.05 a
24	R/B	2.71 ± 0.01 a	35.47 ± 3.02 d	85.37 ± 3.78 b	1.85 ± 0.10 h	2.42 ± 0.27 e	0.72 ± 0.01 d	0.93 ± 0.03 b
	R/B/P	2.52 ± 0.02 c	43.27 ± 3.52 b	67.40 ± 2.00 e	2.43 ± 0.02 f	2.04 ± 0.21 f	0.55 ± 0.02 g	0.72 ± 0.02 e
	R/B/P/G	2.12 ± 0.02 f	36.90 ± 3.46 c	75.60 ± 2.02 c	3.92 ± 0.03 c	1.85 ± 0.26 g	0.63 ± 0.05 f	0.72 ± 0.04 e
	W	2.21 ± 0.02 e	27.17 ± 2.31 h	61.40 ± 1.87 g	1.46 ± 0.03 i	1.56 ± 0.43 h	0.50 ± 0.02 h	0.66 ± 0.05 f
F-test	A	**	**	**	**	**	**	**
	B	**	**	**	**	**	**	**
	A × B	**	**	**	**	**	**	**

^a^ The light qualities are R/B (88.9% red+ 11.1% blue), R/B/P (80.0% red+ 10.0% blue+ 10.0% purple), R/B/P/G (72.7% red+ 9.1% blue+ 9.1% purple+ 9.1% green), W (12.7% red+ 3.9% blue+ 83.4% green)

^b^ mean ± standard deviation, *n* = 3

^c^ Any two means within a column not followed by the same letter are significantly different at *P* < 0.05 by ANOVA on Duncan’s multiple range test

^**^ mean significant at *P* < 0.01

### Effects of photoperiods and light qualities on chlorophyll

Photoperiod and light quality significantly affected chlorophyll a, chlorophyll b, total chlorophyll content and chlorophyll a/b of the leaves of *C. lanceolata* plantlets (Table 2). And there were significant interactions between photoperiods and light qualities. Plantlets exposed to the photoperiod 16 h exhibited the highest chlorophyll a, chlorophyll b, total chlorophyll content and chlorophyll a/b at most light qualities. During photoperiods 8 and 16 h, chlorophyll a, chlorophyll b, total chlorophyll content and chlorophyll a/b were significant higher in the R/B and R/B/P/G treatment compared to those in the R/B/P and W group (*P* < 0.05). The chlorophyll a, chlorophyll b, total chlorophyll content, and chlorophyll a/b reached the highest point in the R/B treatment during photoperiod 24 h, respectively.

**Table 2:** Effects of photoperiods and light qualities on chlorophyll content of *C. lanceolata* in vitro culture plantlets

Photoperiod/ h·d^− 1^ (A)	Light quality^a^ (B)	Chlorophyll a content/mg·g^− 1^ FW	Chlorophyll b content/mg·g^− 1^ FW	Total chlorophyll content/mg·g^− 1^ FW	Chlorophyll a/b
8	R/B	1.02 ± 0.07^b^ d^c^	0.63 ± 0.04 d	1.65 ± 0.12 d	1.64 ± 0.01 d
	R/B/P	0.56 ± 0.03 f	0.40 ± 0.01 f	0.96 ± 0.04 g	1.41 ± 0.03 e
	R/B/P/G	1.43 ± 0.10 a	0.79 ± 0.04 a	2.22 ± 0.14 a	1.81 ± 0.04 ab
	W	0.64 ± 0.02 f	0.47 ± 0.01 e	1.11 ± 0.03 f	1.37 ± 0.01 e
16	R/B	1.30 ± 0.01 b	0.69 ± 0.05 bc	1.99 ± 0.04 b	1.87 ± 0.13 a
	R/B/P	1.17 ± 0.06 c	0.66 ± 0.03 cd	1.83 ± 0.08 c	1.77 ± 0.03 bc
	R/B/P/G	1.35 ± 0.07 ab	0.72 ± 0.02 cd	2.08 ± 0.09 b	1.86 ± 0.07 ab
	W	1.13 ± 0.04 c	0.64 ± 0.01 d	1.77 ± 0.05 cd	1.77 ± 0.02 bc
24	R/B	0.84 ± 0.07 e	0.50 ± 0.03 e	1.33 ± 0.10 e	1.69 ± 0.04 cd
	R/B/P	0.32 ± 0.01 g	0.22 ± 0.01 h	0.54 ± 0.01 h	1.43 ± 0.09 e
	R/B/P/G	0.29 ± 0.02 g	0.26 ± 0.01 g	0.55 ± 0.03 h	1.14 ± 0.03 f
	W	0.34 ± 0.02 g	0.28 ± 0.01 g	0.62 ± 0.03 h	1.20 ± 0.04 f
F-test	A	**	**	**	**
	B	**	**	**	**
	A × B	**	**	**	**

^a^ The light qualities are R/B (88.9% red+ 11.1% blue), R/B/P (80.0% red+ 10.0% blue+ 10.0% purple), R/B/P/G (72.7% red+ 9.1% blue+ 9.1% purple+ 9.1% green), W (12.7% red+ 3.9% blue+ 83.4% green)

^b^ mean ± standard deviation, n = 3

^c^ Any two means within a column not followed by the same letter are significantly different at *P* < 0.05 by ANOVA on Duncan’s multiple range test

^**^ mean significant at *P* < 0.01

### Effects of photoperiods and light qualities on chlorophyll fluorescence

Photoperiod and light quality significantly affected photochemical efficiency of photosystem II (PSII) (Fv/Fo), maximum quantum yield of PSII (Fv/Fm), effective photochemical quantum yield of PSII (Y (II)), photochemical quenching coefficient (qP), non-photochemical quenching coefficient (NPQ/4), and relative electron transport rate in PSII (ETR_II_) of the leaves of *C. lanceolata* plantlets (Table 3). And there were significant interactions between photoperiods and light qualities. Plantlets exposed to the photoperiod 16 h exhibited the higher Y (II), qP, NPQ/4 and ETR_II_ at most light qualities compared to those with photoperiods 8 h and 24 h, but Fv/Fm during photoperiod 16 h was lower than 8 h and 24 h. And the Fv/Fm value of different treatments was between 0.784–0.854. During photoperiods 8 and 16 h, the Fv/Fo, Y(II), qP, and ETR_II_ were significantly greater under the R/B/P/G treatment than R/B, R/B/P and W (*P* < 0.05). On the other hand, there was no significant difference in Fv/Fm between R/B, R/B/P, B/R/P/G and W under photoperiod 16 h.

**Table 3:** Effects of photoperiods and light qualities on chlorophyll fluorescence of *C. lanceolata* in vitro culture plantlets

Photoperiod/ h·d^− 1^ (A)	Light quality^a^ (B)	Fv/Fo	Fv/Fm	Y(II)	qP	NPQ/4	ETR_II_
8	R/B	1.374 ± 0.105^b^ de^c^	0.814 ± 0.009 cd	0.034 ± 0.003 e	0.078 ± 0.004 cde	0.192 ± 0.026 b	2.428 ± 0.220 e
	R/B/P	1.348 ± 0.049 de	0.812 ± 0.005 cde	0.036 ± 0.002 e	0.038 ± 0.003 f	0.234 ± 0.007 a	1.697 ± 0.057 e
	R/B/P/G	1.754 ± 0.037 ab	0.854 ± 0.002 a	0.050 ± 0.005 cd	0.095 ± 0.007 bc	0.171 ± 0.007 ab	5.074 ± 0.638 bc
	W	1.402 ± 0.079 de	0.803 ± 0.005 def	0.050 ± 0.00 cd	0.072 ± 0.006 de	0.164 ± 0.017 de	3.533 ± 0.153 d
16	R/B	1.283 ± 0.070 e	0.792 ± 0.013 ef	0.035 ± 0.004 e	0.062 ± 0.001 e	0.217 ± 0.010 a	3.241 ± 0.236 d
	R/B/P	1.300 ± 0.092 de	0.788 ± 0.010 f	0.035 ± 0.002 e	0.064 ± 0.010 e	0.168 ± 0.011 cde	3.413 ± 0.250 d
	R/B/P/G	1.422 ± 0.051 de	0.784 ± 0.018 f	0.064 ± 0.003 ab	0.138 ± 0.014 a	0.151 ± 0.013 e	6.585 ± 0.265 a
	W	1.287 ± 0.028 e	0.802 ± 0.014 def	0.059 ± 0.006 bc	0.102 ± 0.003 b	0.220 ± 0.008 a	5.711 ± 0.575 b
24	R/B	1.439 ± 0.050 d	0.792 ± 0.016 ef	0.040 ± 0.005 de	0.077 ± 0.004 de	0.150 ± 0.010 e	4.817 ± 0.324 c
	R/B/P	1.573 ± 0.015 c	0.826 ± 0.014 bc	0.043 ± 0.004 de	0.031 ± 0.023 f	0.189 ± 0.021 bc	4.344 ± 0.820 c
	R/B/P/G	1.691 ± 0.072 bc	0.843 ± 0.002 ab	0.071 ± 0.007 a	0.086 ± 0.009 bcd	0.161 ± 0.019 de	7.311 ± 0.019 a
	W	1.854 ± 0.162 a	0.843 ± 0.014 ab	0.048 ± 0.004 d	0.097 ± 0.008 b	0.179 ± 0.017 bcd	4.933 ± 0.379 bc
F-test	A	**	**	**	**	**	**
	B	**	**	**	**	**	**
	A × B	**	**	*	**	**	**

^a^ The light qualities are R/B (88.9% red+ 11.1% blue), R/B/P (80.0% red+ 10.0% blue+ 10.0% purple), R/B/P/G (72.7% red+ 9.1% blue+ 9.1% purple+ 9.1% green), W (12.7% red+ 3.9% blue+ 83.4% green)

^b^ mean ± standard deviation, *n* = 3

^c^ Any two means within a column not followed by the same letter are significantly different at *P* < 0.05 by ANOVA on Duncan’s multiple range test

^*^, ^**^ mean significant at 0.01 < *P* < 0.05 and *P* < 0.01, respectively

### Relationship between growth and chlorophyll fluorescence parameters

Among environmental conditions, light is a predominant source of energy for plant photosynthesis and also an important signal for plant growth and development [20]. Studying the relationship between plant growth and chlorophyll fluorescence under LED can preliminarily reveal the response mechanism of plantlets to light environment. So, we used the growth parameters (plant height, dry weight, rooting rate, average root, root length, root surface area and root volume) as the dependent variable, and the chlorophyll a, chlorophyll b, total chlorophyll content, chlorophyll a/b and chlorophyll fluorescence parameters (Fv/Fo, Fv/Fm, Y(II), qP, NPQ/4, ETR_II_) as independent variables for multivariate stepwise regression analysis. The results (Table 4) showed that Fv/Fm, Y(II), and ETR_II_ were significant correlated with plant height (*P* < 0.001), while the chlorophyll and chlorophyll fluorescence parameters was not correlated with dry weight. ETR_II_ and total chlorophyll content were significant correlated with rooting rate (*P* < 0.001); Fv/Fo and ETR_II_ (*P* < 0.001) were significant correlated with average root number; Fv/Fm, ETR_II_, and total chlorophyll content were significant correlated with root length (*P* < 0.001); Fv/Fm and ETR_II_ were significant correlated with root surface area (*P *< 0.001); ETR_II_, total chlorophyll content, and chlorophyll a/b ratio were significant correlated with root volume (*P *< 0.001).

**Table 4:** Linear regression model of plant growth parameters and chlorophyll, chlorophyll fluorescence parameters

Growth parameters	Linear regression model	AIC	R^2^	P
Plant height	y = 8.028–7.074 × _1_–34.508 × _2_ + 0.359 × _3_	−30.584	0.882	0.000
Rooting rate	y = − 46.617 + 11.847 × _3_ + 38.395 × _4_	77.915	0.796	0.001
Average root	y = 5.379 + 0.867 × _3_–4.520 × _5_	9.754	0.809	0.001
Root length	y = 25.812–36.655 × _1_ + 0.687 × _3_ + 2.123 × _4_	7.718	0.856	0.001
Root surface area	y = 12.271–15.441 × _1_ + 0.232 × _3_	−13.118	0.787	0.001
Root volume	y = −1.574 + 0.129 × _3_ + 1.418 × _4_–0.401 × _6_	−33.983	0.861	0.001

*AIC* Akaike information criterion, *R*^*2*^ R Square, *x*_*1*_ Fv/Fm, *x*_*2*_ Y(II), *x*_*3*_ ETR_II_, *x*_*4*_ Total chlorophyll content, *x*_*5*_ Fv/Fo, *x*_*6*_ Chlorophyll a/b.

### Principal component analysis

**Fig. 2:**
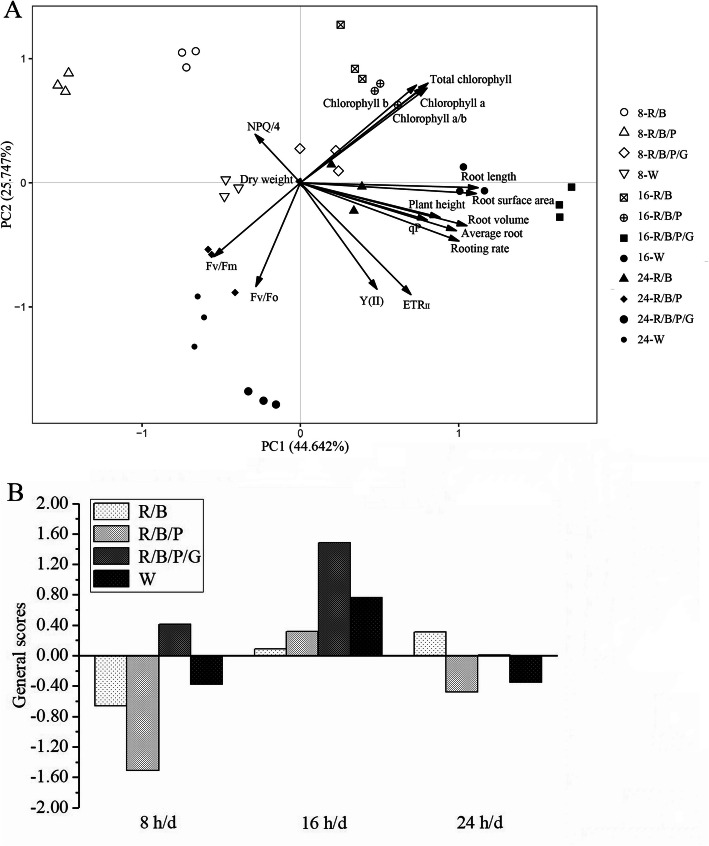
Principal component analysis of 17 parameters of the *C. lanceolata* in vitro culture plantlets under different LED treatments. **a** PCA biplot. **b** Comprehensive score of LED photoperiod, light quality treatments

The principal component analysis of 17 parameters of the *C. lanceolata* in vitro culture plantlets under different LED treatments was conducted. The results showed that the eigenvalues of the first four principal components, PC1, PC2, PC3, and PC4 were all greater than 1, and the cumulative contribution rate reached 89.927%, indicating that these four principal factors could be used to explain 89.927% of the variation in our data (Table 5, Fig. 2a). The first principal component PC1 could explain 44.642% of the total variation, which was closely related to the original variables of rooting rate, average root, root length, surface area, and volume (R = 0.829–0.939), reflecting the root morphology of the plantlets. The second principal component PC2 can explain 25.747% of the total variation, which was closely related to the original variables of Fv/Fo, Y(II), ETR (R = 0.731–0.757), reflecting the chlorophyll fluorescence of the plantlets. The third component PC3 can explain 12.479% of the total variation, which was negatively related to the original variable of dry weight (R = -0.780), reflecting the accumulation of organic matter in the plantlets. The fourth principal component PC4 can explain 7.059% of the total variation, which was negatively related to the original variable of NPQ/4 (R = -0.473), reflecting the heat dissipation of the excitation energy of the seedling antenna pigment system.

**Table 5:** Rotated component matrix of PCA

Factor	Principal components			
	PC1	PC2	PC3	PC4
Plant height	0.743	0.205	−0.516	0.213
Dry weight	0.020	0.024	−0.780	0.423
Rooting rate	0.849	0.383	−0.051	0.161
Average root	0.829	0.293	−0.158	− 0.248
Root length	0.939	0.002	−0.159	−0.198
Root surface area	0.931	0.044	−0.096	−0.237
Root volume	0.885	0.262	−0.242	0.067
Chlorophyll a	0.653	−0.643	0.318	0.210
Chlorophyll b	0.598	−0.663	0.394	0.181
Total chlorophyll	0.665	−0.688	0.049	0.209
Chlorophyll a/b	0.637	−0.650	0.343	0.201
Fv/Fo	−0.235	0.751	0.330	0.453
Fv/Fm	−0.474	0.560	0.448	0.300
Y(II)	0.431	0.731	0.300	−0.319
qP	0.700	0.257	0.575	−0.077
NPQ/4	−0.380	−0.478	− 0.020	−0.473
ETR_II_	0.605	0.757	0.137	−0.003
Characteristic root	8.396	1.147	0.872	0.863
Contribution rate	44.642%	25.747%	12.479%	7.059%
Cumulative contribution rate	89.927%

The quality of the *C. lanceolata* in vitro culture plantlets could be determined according to the comprehensive scores of PC1, PC2, PC3, and PC4. As shown in Fig. 2b, the comprehensive score of the photoperiod 16 h was significantly higher than photoperiods 8 h and 24 h. During photoperiod 16 h, the light quality R/B/P/G treatment had the highest comprehensive score of 1.480, indicating that 16-R/B/P/G treatment was the best LED illumination condition for the growth of the *C. lanceolata* in vitro culture plantlets.

## Discussion

### Growth

Photoperiod can not only affect plant flowering induction and flower differentiation, but can also affect its vegetative growth and physiological and biochemical characteristics [21, 22]. Plants can sense subtle changes in light quality through photoreceptors, which in turn regulate growth and development of plants through exciting signaling pathways [23]. In the present study, we found that plantlets during photoperiod 16 h showed higher height, dry weight, rooting rate, average root number, root length, root surface area, and volume at most light qualities compared to photoperiods 8, 24 h. And the best plantlets were harvested with more roots and leaves. Similar results were reported by Li et al. [24], the plantlet growth and root growth of *Acacia melanoxylon* found to be the highest among in vitro culture under photoperiod 16 h. Plants mainly perceive light signals through photoreceptors such as red/far red light receptors, blue/near ultraviolet photoreceptors, and ultraviolet photoreceptors, to regulate plant growth and physiological metabolism [19, 25]. Our results showed that plant height, rooting rate, root length, root surface area and root volume of R/B/P/G were higher than R/B, R/B/P and W under photoperiods 8 and 16 h, while they were lower in the R/B, R/B/P than W, suggesting that the red-blue-purple-green composite light could increase growth of *C. lanceolata* in vitro culture plantlets. This may be due to that red and blue light are the most effectively utilized wavelengths for plants photosynthesis as absorption spectrum of photosynthetic pigments mainly focus on red light spectrum (600–700 nm) and blue (400–500 nm). Among them, red light can regulate the operation of the plant photosynthetic system and the transport of assimilates [26]; blue light is involved in stomatal opening [27]; and green light can penetrate into the plant canopy for its higher reflectivity and transmittance compared with other wavelengths lights [28]. Furthermore, phytochromes and cryptochromes are receptors not only for red and blue light, but also for purple and green light, which might explain why red light combined with blue, purple and green light (72.7% red+ 9.1% blue+ 9.1% purple+ 9.1% green) positively affects height and root growth of *C. lanceolata* plantlets cultured in vitro.

### Chlorophyll

Chlorophyll is vital for photosynthesis, which allows plants to absorb energy from light. The ratio of chlorophyll a/b can reflect the change of photosystem, relating to the PSII:PSI content and the size of PSII light-harvesting antenna [29, 30]. The higher level of chlorophyll a/b suggests the stronger light adaptability, the higher electron transport ability of chlorophyll and the higher activity of Calvin cycle enzyme [31]. The results of the present experiment showed that photoperiod 16 h treatment promoted the accumulation of chlorophyll a, chlorophyll b, total chlorophyll, and the increase of chlorophyll a/b ratio more than other treatments examined. Our results suggested that the photoperiod 16 h could promote the ability of leaves of *C. lanceolata* in vitro culture plantlets to convert, capture and transfer light energy more efficiently compared to photoperiods 8, 24 h. Neither extremely short nor long photoperiods increase the chlorophyll content and the chlorophyll a/b ratio. In addition, we also showed that chlorophyll a, chlorophyll b, total chlorophyll content, and chlorophyll a/b ratio under R/B/P/G treatment were higher than those under R/B/P treatment, indicating that the effect of red light combined with blue, purple light was not as best compared to the red-blue-purple-green composite light, because the gain effect of composite light was not a mere accumulation of monochromatic light, but the result of the interaction between spectrum and plant spectral pigment system [32, 33]. This result reflects Guo et al.’ [34] finding that *Paeonia suffruticosa* Andr. plantlets had higher chlorophyll content under red-blue-yellow-green-purple composite light than red-blue, red-blue-yellow, red-blue-green, and red-blue-purple composite light.

### Chlorophyll fluorescence

Chlorophyll fluorescence, a nonintrusive indicator for rapid assessment of in vivo photosynthesis, is widely used in the study of the effects of different environmental conditions on plant photosynthesis. The process of light energy absorption, utilization, transfer and dissipation of PSI and PSII (mainly PSII) can be reflected by the change of chlorophyll fluorescence parameters [35]. Several studies showed that light quality has significantly effect on chlorophyll fluorescence parameters in leaves of plantlets, such as cucumber (*Cucumis sativus* L.) plantlets exposed to R/B 8:1 treatment significantly increased electron transport rate in PSII (ETR_II_) and in PSI (ETR_I_) by 176.9% and 127.0%, respectively, compared with the R treatment [36]. Wang Y et al. [37] also found that the Fv/Fm, qP and ETR_II_ of *Dendrobium candidum* plantlets under red and blue compound light were higher than monochrome red light. Fv/Fm is an important indicator to measure the degree of photoinhibition and can be used to indicate the level of PSII primary light energy conversion efficiency [38]. Previous studies have shown that the Fv/Fm of most healthy vascular plants is 0.832 [39]. If the value is greatly reduced, it means that the plant is subject to photoinhibition and the reaction center is irreversibly damaged or reversibly inactivated [38, 40]. Although the Fv/Fm at photoperiod 16 h was lower than that at 8 h and 24 h in our study, the variation was small (0.784–0.854), indicating that the *C. lanceolata* in vitro culture plantlets were not subjected to light inhibition or stress. Y(II) represents the part of energy used for photochemical reaction after absorption of PSII [41]. qP and NPQ reflect the share of light energy absorbed by PSII antenna pigments that can be used for photochemical electron transfer and the share dissipated in the form of heat, respectively [38, 42, 43]. In this study, Y(II), qP, NPQ/4 and ETR_II_ at photoperiod 16 h were higher than those indicators at 8 and 24 h under most treatments of light combinations, suggesting that the light energy absorbed in the leaves of *C. lanceolata* in vitro culture plantlets at photoperiod 16 h treatments can be good used for photosynthetic electron transportation. Furthermore, the above changes might indicate that PSII function enhanced and excitation energy from reaction center increased, which was conducive to improving photosynthetic efficiency. We also found that Fv/Fo, Y(II), qP, and ETR_II_ under R/B/P/G were higher than R/B, R/B/P and W during photoperiods 8 and 16 h, which indicated that PSII reaction center under R/B/P/G opened more than that under R/B, R/B/P and W, and absorbed more light energy for photosynthesis to a greater extent, and the plantlets have higher electron transfer efficiency and stronger photosynthetic capacity. It may be the reason that plantlets have higher height and greater leaves and roots under R/B/P/G light treatment.

### Comprehensive analysis

Previous studies on in vitro culture of *C. lanceolata* mainly focused on adjusting the basic growth medium. Most of the light sources were fluorescent and incandescent lamps. The rooting rate of plantlets was about 30–70% and the average root number was about 2–4 roots. In addition, the best culture time-period is more than 40–45 days [16]. Our results demonstrated that 16-R/B/P/G was best for growing *C. lanceolata* plantlets in vitro. Under this treatment, the rooting rate and the average root number were 95.50% and 4.45, respectively, and the culture time was 30 days. The root length, root surface area and root volume could reach the highest. The results showed that we could shorten the culture time, increase the rooting rate, and harvest the best *C. lanceolata* plantlets with more leaves and roots, by using appropriate LED photoperiod and light quality. This indicated that photoperiod 16 h and light quality R/B/P/G (72.7% red+ 9.1% blue+ 9.1% purple+ 9.1% green) made the shoots and roots growth of *C. lanceolata* in vitro culture plantlets superior to other treatments, and the best synergistic effect on various signal receptors of light signal reception, recognition, transmission and response was produced. We also found that total chlorophyll content and ETR_II_ were significant correlated with rooting rate, root length and root volume, while Fv/Fm and ETR_II_ were significant correlated with plant height, average root number and root surface area, indicating that there were correlation between the growth of *C. lanceolata* in vitro culture plantlets and the chlorophyll and chlorophyll fluorescence of their leaves under different photoperiod and light quality. This may be because higher plants have a set of fine light receiving system and light signal transduction system, which can detect the changes of photoperiod and light quality and make adaptive response. Photoperiod and light quality may affect the growth of shoots and roots of *C. lanceolate* in vitro culture plantlets through two ways: one is photosynthesis through pigment system, the other is a series of physiological and biochemical reactions caused by light signal received by photoreceptor [44]. Light may be related to gene expression of carbon and nitrogen metabolism pathway [45]. The changes of light conditions (including photoperiod and light quality) may induce the regulation of photosensitive pigments on sucrose metabolizing enzymes, increase the activities of related sucrose metabolizing enzymes, and make the plantlets accumulate more photosynthates [46]. In addition, photosynthetic carbon metabolism provides energy and carbon framework for nitrogen metabolism [47], which is beneficial for the plantlets to synthesize amino acids and proteins, etc. Although light does not directly affect the growth of plant roots, it can affect root morphogenesis and growth by affecting photosynthesis and the synthesis and transport of photosynthates, but the mechanism needs to be further explored.

## Conclusions

The growth, chlorophyll and chlorophyll fluorescence of *C. lanceolata* in vitro plantlets were found to be significantly affected by photoperiods, light qualities and their interactions. In vitro cultured plantlets subjected to photoperiod 16 h had longer root, higher height, rooting rate, root number, and the higher levels of chlorophyll, chlorophyll a/b, Y(II), qP, NPQ/4 and ETR_II_ compared to photoperiods 8 h and 24 h, but Fv/Fm during photoperiod 16 h was lower than 8 h and 24 h. On the other hand, plantlets exposed to R/B/P/G generated more root and presented higher chlorophyll, Fv/Fo, Y(II), qP, and ETR_II_ than W during photoperiods 8 and 16 h. Total chlorophyll content and ETR_II_ were significant correlated with rooting rate, root length and root volume, while Fv/Fm and ETR_II_ were significant correlated with plant height, average root number and root surface area. 16-R/B/P/G is best for growing *C. lanceolata* plantlets in vitro.

## Methods

### Plant material

The present experiment was conducted between March and June in 2016. The 12th generation in vitro plantlets of *C. lanceolata* used in this study were provided by the College of Forestry at Guangxi University in China. The plant materials were formally identified by Lecturer Rongyan Deng and deposited in the laboratory of College of Forestry at Guangxi University. The plantlets were pretreated as described by Xu et al. [48]. Subsequently, 540 healthy unrooted shoots with consistent growth (approximately 1.5 ± 0.2 cm height) were applied to further LED experiments.

### Experimental design

The “T5 2835 LED plant tube” produced by Shenzhen Weixinli Optoelectric Co., Ltd. was used as light source for cultivation. The size of the lamp is 1200 mm × 24 mm, and the rated power is 16 W. Each lamp consists of 96 sets of 2835 LED chips produced by San’an Optoelectronics. The lamp bead is evenly arranged in different color proportions, the rated voltage is AC 85–265 V, the working current is 100 mA and the frequency is 50/60 Hz. The peaks of emission of different LED lights are for red light at 620–630 nm, 460–470 nm for blue light, 410–420 nm for purple light, and 520–530 nm for green light. We used three photoperiods of 8 h/d (8:00–16:00), 16 h/d (8:00–24:00), and 24 h/d, and under each photoperiod set three composite lights of 88.9% red+ 11.1% blue (R/B), 80.0% red+ 10.0% blue+ 10.0% purple (R/B/P), 72.7% red+ 9.1% blue+ 9.1% purple+ 9.1% green (R/B/P/G), and white light (12.7% red+ 3.9% blue+ 83.4% green, W) was used as control. There were 12 treatments in the experiment, with 3 replications per treatment. Forty-five plantlets were used per replication, and divided into 15 bottles (3 plantlets per bottle), and cultured for 30 d. Three replications of each treatment were placed in a separate chamber, which was separated by a dark cloth. According to the conclusion that photosynthetic photon flux density (PPFD) 30 μmol·m^− 2^·s^− 1^ blue light was the best for chlorophyll increased in vitro culture of *C. lanceolata* by Zhou et al. [18], we used two light intensities (20, 30 μmol·m^− 2^·s^− 1^) with red blue 4:1 to screen the best light intensity for the growth of *C. lanceolata* cultured in vitro. The results showed that rooting rate, average root number and root length under 20 μmol·m^− 2^·s^− 1^ were higher than 30 μmol·m^− 2^·s^− 1^ (Unpublished data). So, 20 μmol·m^− 2^·s^− 1^ was used in this study. Two corresponding lamps were used in each chamber, and the distance between the light source and the plant was adjusted accordingly so that PPFD was constant (about 20 ± 5 μmol·m^− 2^·s^− 1^ for all treatments at plant height, approximately a vertical distance of 2.5 cm from the culture shelf where the culture bottles were placed). PPFD was measured using a quantum sensor (LI-250A; LiCor Inc., Lincoln, NE, USA). All the cultures were incubated in a controlled environment at 25 ± 1 °C and 60 ± 5% relative humidity.

### Growth

The total number of plantlets, rooting plantlets, and roots were counted and the rooting rate and average number of roots were calculated (see below). Ten plantlets were randomly selected for each replication for determining the root length, surface area, and volume with an Epson automatic root scanning analyzer (Seiko Epson Corporation, Nagano, Japan). Data was analyzed using a Win RHIZOC Pro 2004 b software. The calculation formula is as follows:

- Rooting rate = number of rooting plantlets /total number of plantlets;
- Average number of roots = total number of roots/total number of plantlets;

Ten plantlets were randomly selected for each replication to determine the plant height with a ruler (accuracy of ±0.1 cm). Ten complete plantlets per replication were placed at 105 °C and dried in an oven at 80 °C for 48 h until a constant weight was reached. The dry weight of the plants was determined using an electronic balance (accuracy ±0.1 mg).

### Chlorophyll

Chlorophyll content was estimated according to Porra et al. [49]. We randomly selected 1 g (fresh weight, FW) leaves from each replication were homogenized in a pre-chilled mortar using 20 mL of a 1:1 (v/v) mixture of 80% cold acetone and absolute ethanol. The homogenate was transferred to a 25 mL test tube, centrifuged at 4500 rpm for 10 min, and the supernatant collected. The absorbance was measured with Lambda 35 UV Vis spectrometer (Perkin Elmer, USA) at 663 nm (OD_663_) and at 645 nm (OD_645_) for chlorophyll a and chlorophyll b. The chlorophyll content was determined using the following formulas:

$$ \left(\mathrm{Chlorophyll}\right)\ \mathrm{a}\ \left( mg\cdotp {g}^{-1}\right)=\frac{\left(12.72\times {OD}_{663}-2.59\times {OD}_{645}\right)\times V}{1000\times W} $$

$$ \left(\mathrm{Chlorophyll}\right)\ \mathrm{b}\ \left( mg\cdotp {g}^{-1}\right)=\frac{\left(22.88\times {OD}_{645}-4.67\times {OD}_{663}\right)\times V}{1000\times W} $$

Total chlorophyll content (mg·g^−1^) = chlorophyll a + chlorophyll b

Where V is the total volume of acetone-ethanol extract (mL) and W is FW (g) of sample.

### Chlorophyll fluorescence

Ten plantlets were randomly selected from each replication in every treatment group and placed in a dark room to adapt for 30 min before measuring the chlorophyll fluorescence parameters by a M-series-modulated chlorophyll fluorescence imaging system MAXI-IMAGING-PAM (Heinz Walz GmbH, Effe ltrich, Germany) at room temperature (25 ± 2 °C) in 9:00–12:00 a.m. The software Imaging-Win was used to control MAXI-IMAGING-PAM measuring systems to calculate the chlorophyll fluorescence parameters. The measurement was conducted using the software’s standard procedures. After shading, 3 complete plantlets were randomly selected from 10 and quickly put into the adapter IMAG-MAX/GS (10 × 13 cm) of MAXI-IMAGING-PAM. Ten Area of Interest (AOI) were selected evenly on the leaves of these three plantlets by the software for measuring chlorophyll fluorescence parameters. Minimum fluorescence (Fo) and maximum fluorescence (Fm) yields were measured at a weak modulated measuring beam (< 0.5 μmol·m^− 2^·s^− 1^) and a saturating pulse of light (2700 μmol·m^− 2^·s^− 1^ for 0.8 s) at 1 Hz, respectively. The fluorescence kinetics was induced by turning on the photochemical light (110 μmol·m^− 2^·s^− 1^) for 5 min, and the maximum fluorescence under light adaptation (Fm′) and the actual fluorescence (F) were measured by turning on the saturating pulse every 20 s. All relevant fluorescence parameters, including photochemical efficiency of photosystem II (PSII) (Fv/Fo), maximum quantum yield of PSII (Fv/Fm), effective photochemical quantum yield of PSII (Y(II)), photochemical quenching coefficient (qP), non-photochemical quenching coefficient (NPQ/4), and relative electron transport rate of PSII (ETR_II_) were given automatically by the instrument. Three plantlets were measured per replication as mentioned above, and per measurement was repeated thrice. The mean of the three measurements was the value of the chlorophyll fluorescence parameters of each replication.

### Data analysis

One-Way ANOVA and Duncan multiple comparisons (*P* < 0.05 for significant differences) were used to analyze the differences of each experimental data (Supplementary file 1). Two-Way ANOVA was used to analyze the variance of all experimental data. Meanwhile, the relationship between growth parameters and chlorophyll fluorescence parameters was analyzed by Multiple Stepwise Regression Analysis. The above analysis was performed by SPSS software version 21.0 (IBM Inc., Chicago, IL, USA). Principal Component Analysis (PCA) was used to evaluate the all parameters and to select the best combination of LED photoperiod and light quality for in vitro culture plantlets of *C. lanceolata*. The histogram and biplot were plotted using Origin 2017 SR2 software (OriginLab Inc., Hampton, MA, USA) and R v3.6.2 with the package “ggplot2”, respectively.

## Supplementary information

**Additional file 1.**

## Data Availability

All data generated or analysed during this study are included in this published article and its supplementary information files.

## Abbreviations

LEDs: Light emitting diodes
R/B: Red-blue light;
R/B/P: Red-blue-purple light
R/B/P/G: Red-blue-purple-green light
W: White light
Fv/Fo: Photochemical efficiency of photosystem II (PSII)
Fv/Fm: Maximum quantum yield of PSII
Y (II): Effective photochemical quantum yield of PSII
qP: Photochemical quenching coefficient
NPQ/4: Non-photochemical quenching coefficient
ETR: Relative electron transport rate in PSII
PC: Principal components
PCA: Principal Component Analysis
